# Physical Parameters and Fatty Acids Profiles in Milanino, Mericanel Della Brianza, Valdarnese Bianca and Commercial Hybrids (*Gallus Gallus Domesticus*) Table Eggs

**DOI:** 10.3390/ani10091533

**Published:** 2020-08-31

**Authors:** Stefano Paolo Marelli, Luisa Zaniboni, Manuela Madeddu, Ahmad Abdel Sayed, Maria Giuseppina Strillacci, Maria Grazia Mangiagalli, Silvia Cerolini

**Affiliations:** 1 Department of Veterinary Medicine, University of Milan, via dell’Università 6, 26900 Lodi LO, Italy; luisa.zaniboni@unimi.it (L.Z.); ahmad.abdel@unimi.it (A.A.S.); maria.strillacci@unimi.it (M.G.S.); grazia.mangiagalli@unimi.it (M.G.M.); silvia.cerolini@unimi.it (S.C.); 2 Freelance Veterinarian, 21047 Saronno-Va, Italy; manuelamade@hotmail.com

**Keywords:** Mericanel della Brianza chicken breed, Milanino chicken breed, Valdarnese Bianca chicken breed, hybrid strains, commercial hybrid strains, egg quality

## Abstract

**Simple Summary:**

Conservation of traditional poultry breeds is closely linked to product valorization considering the renewed interest in local poultry breeds production and consumers’ orientation towards food items considered healthier and safer. The aim of the present research is to investigate the differences in egg physical parameters and fatty acid profile of three traditional chicken breeds (Mericanel della Brianza, Milanino and Valdarnese Bianca) and two commercial hybrids using marked-procured eggs. We evaluated the effects of the breeds and of the genetic origin (traditional breed or hybrid strain) on egg physical and chemical parameters, furthermore we analyzed the most influencing parameters and their effects on egg groups differentiation via Principal Component Analysis (PCA). Eggs produced by traditional breeds differentiate from eggs produced by commercial hybrids in physical and fatty acids parameters. The nutritional value of eggs obtained from traditional breeds has been demonstrated to be higher considering the yolk content, the Polyunsatured Fatty Acids - PUFA fraction, the n6/n3 ratio and the atherogenic and thrombogenic indexes. Commercial layers’ eggs revealed their higher commercial value based on weight, albumen content and percentage of edible content.

**Abstract:**

The aim of the present study is to investigate the physical parameters and fatty acid composition and related nutritional parameters of market-procured table eggs from Milanino, Mericanel della Brianza and Valdarnese Bianca hens compared to two commercial hybrid strains’ eggs to determine characterizing quality traits for traditional breeds conservation and valorization through high quality niche products. Fifty-four market eggs by three traditional breeds (Mericanel della Brianza—MRC; Milanino—MLN; and Valdarnese Bianca—VLD) and two commercial hybrid strains (Commercial Hybrid Brown—CHB; Commercial Hybrid White—CHW) have been analyzed—physical parameters, fatty acids profile and atherogenic and thrombogenic indexes were investigated. A General Linear Model—GLM was applied to data analysis with breed and genetic origin (traditional breed—TRD; commercial hybrid—HYB) as sources of variation. Two Principal Component Analyses (PCA) were carried out with physical parameters and fatty acid parameters as variables. Eggs produced by traditional breeds MRC MLN and VLD differentiate from eggs produced by commercial hybrids CHB and CHW in physical and chemical parameters (fatty acids parameters). The nutritional value of the traditional eggs has been demonstrated to be higher considering the yolk content, the PUFA fraction, the more favorable n6/n3 ratio and the atherogenic and thrombogenic indexes. Commercial layers’ eggs revealed their higher commercial value based on weight, albumen content and percentage of edible content.

## 1. Introduction

Animal production has been characterized, in the past century, by a strong intensification of production systems and a dramatic reduction of traditional breeds. Traditional breeds well adapted after centuries of natural and artificial selection in specific geographic and climatic areas have been replaced with highly productive synthetic strains or commercial hybrids [1,2].

Two main sectors can be identified in the global poultry production—the large scale production led by multinational corporations based on highly productive hybrid strains and the small scale production characterized by a variety of traditional breeds and populations (predominant in developing countries) [2]. In developed countries the conservation of poultry genetic diversity is mainly based on the creation of genetic reservoirs to preserve and optimize global poultry production [3,4,5]. Furthermore, the inclusion of specific characterizing properties (special traits) and the cultural value of the breeds must be taken into consideration [6].

Traditional breeds and local populations are characterized by specific genetic backgrounds enabling them to adapt to specific conditions and to produce valuable products [1]. Product characterization is fundamental in value estimation—in animal products in particular, the nutritional quality is based on proteins and lipids composition together with macro and trace elements profile and the presence of allergens [7]. Fatty acids profile is an additional trait in animal products’ quality determination [8]. Consumers’ perception of animal product quality is oriented to extrinsic ‘on farm’ aspects too like production system and facilities, feed origin and transport. In addition traceability, farm assurance protocols and official certifications (organic label) are considered effective in product differentiation [9]. Traditional breeds are indicated to be used for organic production in European legislation (Council Regulation 2007/834/EC). Traditional breeds are considered to be more adapted to extensive production systems with lower environmental control and input than commercial hybrid strains [10]. *Gallus gallus domesticus* egg physical parameters and fatty acid profile have been widely demonstrated to be discriminating genetic-based factors in breed differentiation [10,11,12,13,14,15]; however little is known about heritage breeds’ egg fatty acid profiles despite consumers’ perception that these eggs are healthier product as compared with eggs from commercial breeds [12].

Mericanel della Brianza, Milanino and Valdarnese Bianca breeds are autochthonous Italian breeds from the Lombardy region (northern Italy; Milanino and Mericanel) and from the Tuscany region (central Italy; Valdarnese Bianca). Milanino and Valdarnese Bianca are mainly considered meat type traditional breeds, Mericanel della Brianza is mainly known for its brooding ability. No data are available about the characteristics of the table eggs from these breeds.

The aim of the present study is to investigate the physical parameters and fatty acid composition and related nutritional parameters of table eggs from Mericanel della Brianza, Milanino and Valdarnese Bianca hens compared with two commercial hybrid strains’ eggs. These parameters are used to characterize quality traits for traditional breeds conservation and valorization through high quality niche products. All eggs were purchased from commercial sources.

## 2. Materials and Methods

### 2.1. Eggs Breeds and Strains

Fifty-four market eggs from three traditional breeds (Mericanel della Brianza—MRC; Milanino—MLN; and Valdarnese Bianca—VLD) and two commercial hybrid strains (Commercial Hybrid Brown—CHB; Commercial Hybrid White—CHW) were analyzed. Birds’ breeds, samples distribution, genetic origin, egg shell color, hen weight range and official recognition by the official poultry club recognized by Agriculture Ministry FIAV (Federazione Italiana Associazioni Avicole) are listed in Table 1. Eggs were purchased in farms’ shops.

### 2.2. Physical Parameters

Physical parameters were recorded by individually weighing (Sartorius Analytic A200S, Goettingen, Germany) the whole egg (WEG), the yolk (YOL; separated with a yolk separation cup for cooking use), the albumen (ALB) and the shell (SHE; membranes included). Edible part (yolk + albumen), albumen, yolk, shell percentage and edible fraction percentage were calculated on the whole egg weight.

### 2.3. Fatty Acid Profile

The total lipids were extracted from singular yolk samples in a suitable excess of chloroform/methanol (2:1, vol.:vol.) [16,17]. Fatty acids were trans-methylated by refluxing in methanol: toluene: sulphuric acid (20:10:1, vol.:vol.:vol.) in the presence of pentadecanoic acid standard [18]. Fatty acid quantification was obtained by gas chromatography by injection via a CP9010 autosampler (Chrompack, Speck Analytical, London, UK) onto a capillary column (Carbowax, 30 m × 0.25 mm, film thickness 0.25 μm; Alltech ltd., Carnforth, UK) in a CP9001 Chrompack gas chromatograph connected to a data processing system (EZ-Chrom data handling system; Speck Analytical, UK). The identification of the peaks was determined by comparison with the retention times of external standard fatty acid methyl ester mixtures. The amount of each fatty acid was calculated comparing fatty acids peaks areas to the peak area of Pentadecanoic fatty acid (standard) [19]. Fatty acids content is presented as fatty acids percent—(fatty acid/total fatty acids) * 100. Only fatty acids with a concentration ≥ 1% have been reported. The proportion of total saturated (SFA), monounsaturated (MUFA) and polyunsaturated fatty acids (PUFA) and the n-6/n-3 ratio were calculated together with atherogenic index (AI) and thrombogenic index (TI) according to Ulbricht and Southgate functions [20].

### 2.4. Statistical Analysis

Statistical analysis was performed by the analysis of variance (ANOVA) using General Linear Model procedure of SPSS (IBM SPSS Statistics, New York, NY, USA) to assess the effect of the breed and of the genetic origin on egg physical parameters and fatty acids profile, the post hoc Bonferroni test was used to investigate the significant differences at breed and genetic origin levels [21]. In order to identify the more influencing variables and to better visualize the distribution of the eggs according to their origin (breed), two Principal Component Analysis (PCA) were carried out with physical parameters and fatty acids parameters as variables. Two scatterplots were produced (PCA physical parameters; PCA fatty acids) [22].

## 3. Results

All physical parameter variables were significantly influenced by the breed of the hen and the genetic origin. Physical parameters are presented in Table 2.

Egg weights differed among the breeds, Mericanel della Brianza (MRC), the only bantam breed, had significantly lighter eggs than those of all other breeds. Valdarnese Bianca (VLD) eggs were significantly lighter then Commercial Hybrid Brown (CHB) and Milanino (MLN) eggs. The genetic origin significantly influenced egg’s weight, eggs produced by commercial hybrids HYB were the heaviest.

Albumen weight was higher in the two hybrids strains than in the three traditional breeds. The highest albumen weight was recorded in CHB birds, the lowest in MRC.

Yolk weight differentiated the breeds and their genetic origin. The highest yolk weight was recorded in Milanino (MLN) eggs. Traditional breeds (TRD) birds produced eggs with a heavier yolk weight.

Eggshell weight was different in the five considered groups—MLN birds produced the heaviest shell. The HYB eggs had heavier shells when compared with all breeds of TRD genetic origin.

The edible part was heavier in the heaviest eggs—CHB eggs, edible fraction weight was higher in HYB eggs than TRD eggs.

Physical parameters percent proportions gave us a clearer definition of the occurring differences within the five groups’ eggs considering the ratios between the different sections of the egg.

Albumen percentage was higher in HYB eggs than in TRD eggs (62.60% vs. 48.38%); CHB eggs had the highest albumen content—63.77%, the lowest percentage was recorded in MRC eggs 42.14%.

Yolk percentage is inversely proportional to albumen percentage—TRD eggs were higher than HYB eggs (36.52% vs. 24.17%). MRC was the top breed for yolk percentage—41.15% almost doubling CHB (22.96%).

Shell percentage is higher in TRD eggs than in HYB eggs (15.09% vs. 13.22%). MRC the highest shell proportion (16.70%) which highly differentiate it from CHB and CHW shell proportion (13.27; 13.14).

The edible part percentage is higher in HYB eggs and in CHW in particular.

**Figure 1:**
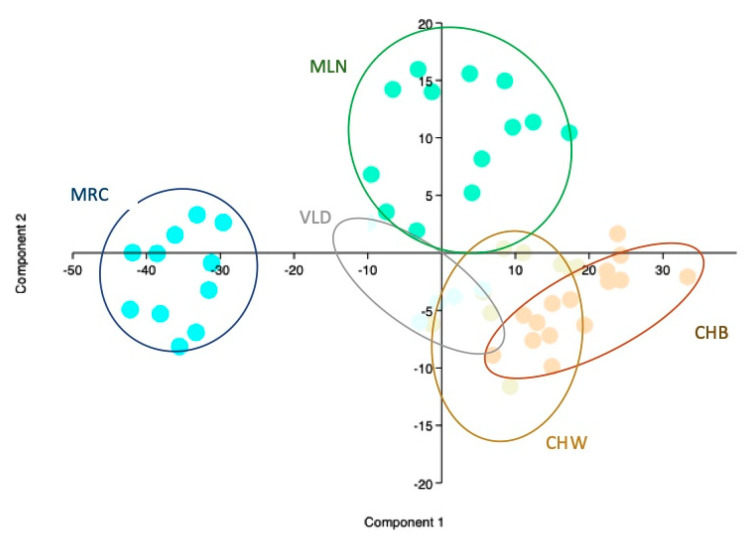
Scatter Plot principal component analysis (PCA) Physical Parameters *. * Every spot represents an egg, every color a breed (CHB = orange, CHW = beige, MLN = green MRC = turquoise, VLD = light blue) breed specific lines circling the eggs of each breed have been defined.

PCA scatter plot for physical parameters is presented in Figure 1. Components 1 and 2 define the 99% of the variance (1 = 88.50%; 2 = 10.34%); egg weight (0.57) and edible part (0.49) were the two leading variables in component 1. In component 2 yolk percentage (0.49) was the determinant variable. A clear differentiation of MLN CHB and CHW eggs on component 1 is presented, CHB and CHW eggs share wide overlapping areas underlining their closeness. MRC and VLD are differentiated on the second component, an evident clustering ability is demonstrated by MRC eggs. Some overlapping between VLD and CHW is present.

A significant effect of the breed on all the detected fatty acids composition in egg yolk was recorded (Table 3). Palmitic acid (C16:0) was significantly the highest in VLD eggs, the same fatty acid was not influenced by birds’ genetic origin. TRD eggs showed a higher content in palmitoleic acid (C16:1n7) than HYB eggs (2.79% vs. 1.10%). The eggs with the highest levels of C16:1n7 were VLD. Stearic acid (C18:0) did not differentiate according to hens’ genetic origin but the breed influenced its content—the highest was recorded in MRC and the lowest in VLD and MLN (8.53). Breed and its genetic origin had a significant effect on oleic acid (C18:1n9). Hybrid hens laid eggs richer in oleic acid than traditional breed hens. Only the breed influences linoleic acid (C18:2n6) content, the highest proportion of linoleic acid have been recorded in CHW eggs and the lowest in VLD eggs. Arachidonic acid (C20:4n6) and docosahexaenoic acid (C22:6n3) were significantly different only in eggs from different breeds, the genetic origin did not influence their content. MRC eggs were the richest in C20:4n-6 and VLD eggs were the richest in C22:6n-3. Birds’ genetic origin did not influence SFA content, the breed did—VLD eggs had the highest SFA level, MLN the lowest. MUFA content was influenced both by breed and genetic origin. HYB eggs were richer in MUFA, at breed level CHB had the highest content in MUFA, MRC the lowest. PUFA content was significantly different by breed and genetic origin—MRC showed the highest content in PUFA and VLD the lowest (24.24 vs. 18.69), at genetic origin level TRD eggs were richer in PUFA than HYB eggs (22.34 vs 20.35). N6/n3 ratio presented significant differences at breed and genetic origin level—Hybrid birds showed a higher value (13.79 vs. 10.91). VLD ratio was the lowest one 5.26, almost one third of CHW n6/n3 ratio (14.51). AI was influenced only by breed with VLD eggs showing the highest value and MRC the lowest. TI was influenced by genetic origin of the birds, white eggs by HYB recording a higher value in TI than TRD (0.96 vs. 0.89). MLN and MRC eggs showed the lowest indexes, CHW and VLD the highest.

**Figure 2:**
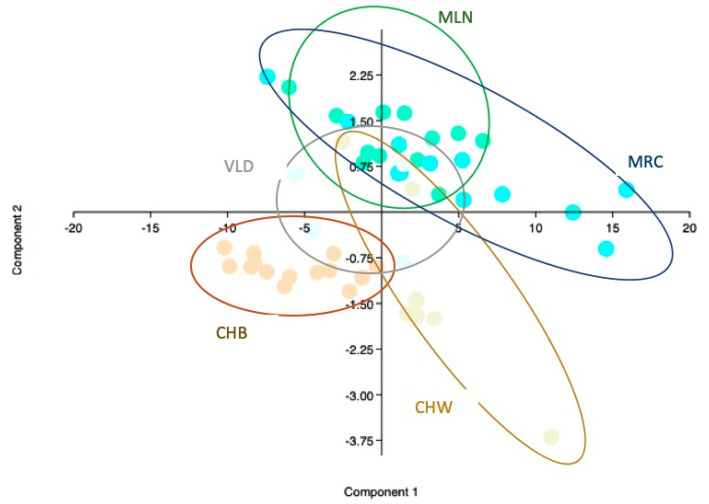
Scatter Plot PCA fatty acids Parameters *. * Every spot represents an egg, every color a breed (CHB = orange, CHW = beige, MLN = green MRC = turquoise, VLD = light blue) breed specific lines circling the eggs of each breed have been defined.

PCA scatter plot for fatty acids parameters is presented in Figure 2. In fatty acids parameters 83% of the variance was defined on the first two components (55.1 and 27.8), PUFA and n6-PUFA were the determining variables on component 1 (0.50, 0.43). On component 2 the most influencing parameter was n6/n3 ratio. Component 1 seemed to differentiate hybrids’ eggs from traditional breeds’ eggs. Within hybrid eggs CHB show a high clustering ability. MLN and MRC cluster together in overlapping areas, once again VLD eggs show overlapping areas with CHW eggs.

## 4. Discussion

Egg physical parameters, fatty acids profile and nutritional values differentiate eggs from traditional breeds and commercial strains. The genetic origin influences egg quality parameters at physical and nutritional levels. Focusing on physical parameters whole egg weight is a basic quality characteristic which regulates egg commercial value according to European Union (EU) legislation about egg grading procedures (2295/2006/CE). CHB and CHW hens laid the heaviest eggs, within the traditional breeds only MLN showed similar weight values. MRC is a bantam breed and so egg weight is clearly lighter. The genetic origin of egg weight is well known together with hen age and with oviposition curve point of lay [14]. Our results (HYB 60.57 g; TRD 48.50 g) are in accordance to those reported by Hocking et al. (2003) who compared 25 different selected and traditional breeds. They described the differences in egg weight between the commercial and the heritage breeds—commercial birds strongly selected for egg weight in the stage of 32 to 35 weeks of age recorded an average egg weight of 63 g vs the 52.5 g of the local layers’ breeds. Differences in average egg weights were recorded in worldwide known standard breeds like Barred Plymouth Rock (56.36 g), White Leghorn (58.36 g), Rhode Island Red (55.95 g) and White Rock (53.60 g) [11], these results are similar to Milanino (MLN) and Valdarnese Bianca (VLD) eggs (59.47 g and 51.91 g). Hanusova et al. (2015) report significant differences between the local Oravka breed and the Rhode Island Red eggs (60.96 g vs. 57.60 g), in this case the local Slovak breed had a weight similar to the Milanino breed [13]. Comparing a brown commercial hybrid and a white commercial hybrid with two local Italian breeds from Veneto Region Robusta Maculata and Ermellinata di Rovigo, the commercial strains showed higher total egg weights both at 30 and 42 weeks of age (Brown 56.9–66.1 g; White 55.5–63.9 g; Robusta 52.5–60.3 g; Ermellinata 51.2–58.8 g) [10]. Two local Italian breeds’ eggs have been recently compared by Di Rosa and colleagues—the Siciliana and the Livorno, the significant difference revealed an heavier weight for Siciliana eggs (54.9 g vs. 48.2 g) [15].

Albumen and albumen content were significantly different in the studied groups—HYB eggs were characterized by an higher proportion of albumen (62.60 vs. 48.38%). Our results are in accordance with those reported by Rizzi and Chiericato (2005) and Hocking et al. (2003), who described the same trend [10,14]. Albumen proportion is correlated to the increase of the greater weight of the individual egg produced by commercial hybrid strains. Selection for egg weight leads to higher albumen content because it is more efficient energetically for hens to synthesize a component with 88% of water compared to the 50% solid yolk [14]. As a consequence yolk weight and, more important, yolk percentage were higher in traditional breeds (36.52% vs. 24.17%). The same results have been described by different authors [10,13,14,23]. The yolk percentage of Mericanel della Brianza eggs took about 41% of the whole egg so the energetic content of the eggs produced by Mericanel dell Brianza hens is significantly higher than the other breeds. Siciliana and Livornese breeds showed results similar to MIL and VLD eggs (32.35 and 30.45) [15]. Hartman in 2000 describes the importance of yolk content for egg processing companies and the need to include this parameter in layers selection protocols [24].

Eggshell and eggshell percentage were significantly higher in TRD breeds; MRC showed the highest eggshell relative weight (16,70%). No significant differences were recorded between CHB CHW and MLN and VDN eggs. Hocking et al. (2003) reported no difference in the relative weight of eggshell from local and commercial breeds [14]. The same results were reported by Rizzi et al. (2006) [25]. Edible part relative weight is higher in HYB eggs, this value could be linked to the high albumen content of these eggs, the nutritional value is mainly based on the lipid fraction which results to be less effectively selected than egg weight–albumen weight-protein fraction [10].

Considering the PCA and the scatterplot in Figure 1 eggs from different breeds showed a perceptible differentiation by physical parameters on the first two principal components much more clearly then by fatty acids parameters. MRC and MLN seemed more clearly differentiated from the other breeds and hybrids. The white and the brown commercial strains showed a clear position on the two components which differentiated them from the traditional breeds except VLD eggs which slightly overlap CHW area. Cluster ability and spatial distribution of the eggs from different breeds and genetic origins are intimately related to commercial parameters like whole egg weight and nutritional parameters like yolk relative weight.

Egg lipids have high nutritional and biological values considering their function as major energy source and as provider of different essential components in embryo development and functionality [8]. Egg yolk fatty acid profile variations may be linked to differences in feed composition, genetic strain, liver physiological reaction (desaturation and elongation) and PUFAs metabolic pathways functionality [26]. Furthermore, PUFAs have specific regulatory functions being involved in the synthesis of a range of biologically active compounds such as eicosanoids [7] and other cell and tissue physiological function regulation as autocrine and paracrine mediators (blood pressure, vasoconstriction and vasodilatation, thrombocyte aggregation, inflammatory reaction and leukocyte activity, bronchial constriction and uterine contractility) [9]. The fatty acids profile in the studied breeds showed high variability with significant differences. Di Rosa et al. (2020) reported between breeds significant differences only for arachidonic acid (C20:4n6) which was higher in Livorno than in Siciliana breed (1.94 vs. 1.64%) [15]. We found similar results in VLD eggs (1.71%) but clearly higher in the other eggs. The only fatty acids which showed significant differences between commercial hybrid birds and traditional breeds are palmitoleic acid (C16:1n7) and oleic acid (C18:1n9) which is the most present fatty acid in hen eggs. TRD eggs showed the highest palmitoleic acid (C16:1n7) content and the lowest oleic acid (C18:1n9) content. Palmitoleic level was similar to those reported in Siciliana and Livornese breeds (2.8; 3.0 and 3.18%) [15]. Oleic acid level in traditional breeds TRD was lower than in Siciliana and Livornese breed, 39% vs. 45% [15]. The highest nutritional value of TRD eggs has been furtherly demonstrated including the high content in PUFA and lower n6/n3 ratio. MRC eggs were the richer in PUFA and VLD eggs were the lower in n6/n3 ratio. Low n6/n3 proportion could be linked to the free range extensive production system which characterize traditional breeds productions [12,27,28]. Atherogenic and thrombogenic indexes were calculated to precisely define the nutritional value of eggs from traditional and commercial breeds on the basis of fatty acid profile [20]. The heathier eggs are those produced by MRC and MLN hens with their lower AI and TI. The values we obtained are similar to those reported by Di Rosa et al. (2020); MLN and MRC eggs are characterized by lower pathogenic risk than both Siciliana’s and Livornese’s eggs [15].

The PCA for fatty acid parameters revealed the 83% of the variance being related to PUFA and n6/n3 proportion. Traditional breeds’ eggs differentiate from HYB eggs on the first component (55.07% of the variance). MRC and MLN eggs are totally overlapping while VLD eggs are close to CHW eggs, similar to the physical parameters’ analysis. Except of MRC and MLN which cluster together VLN, CHW and CHB showed high clustering ability. Fatty acids profile is able, when considering PUFA and n6/n3 ratio in particular to differentiate eggs groups from different breeds and genetic origin.

## 5. Conclusions

In conclusion, market procured eggs produced by Mericanel della Brianza, Milanino and Valdarnese Bianca traditional breeds differentiate from eggs produced by brown and white commercial hybrids under physical and chemical parameters (fatty acids parameters). The nutritional value of the traditional eggs has been demonstrated to be higher considering the yolk content, the PUFA fraction and n6/n3 ratio and the atherogenic and thrombogenic indices. Commercial layers’ eggs revealed their highest commercial value based on weight, albumen content and percentage of edible content. The *per se* value of the traditional breeds is mainly based on adaptation ability and product quality. Objective and effective product characterization procedures could supply helpful tools in genetic reservoir valorization and in breeders’ motivation to produce valuable niche products [1].

## Figures and Tables

**Table 1:** Birds’ breeds, number of samples (*N*), genetic origin (commercial hybrid; traditional breed), egg shell color, hen weight (kg), official registration by Federazione Italiana Associazioni Avicole (FIAV).

Breed	Tag	Samples (N)	Genetic Origin	Shell Color	Hen Weight (kg)	Official FIAV Recognition
*Commercial Hybrid Brown*	CHB	15	Commercial Hybrid	Tinted-brown	1.40–1.48	/
*Commercial Hybrid white*	CHW	9	Commercial Hybrid	White	1.19–1.25	/
*Milanino*	MLN	13	Traditional Breed	Tinted	2.5–3,0	no
*Mericanel della Brianza*	MRC	11	Traditional Breed	Tinted-brown	0.6–0.7	yes
*Valdarnese Bianca*	VLD	6	Traditional Breed	White	2.0–2.5	no

**Table 2:** Physical egg parameters (LS mean ± SE) by breed.

Egg Parameters	Breeds					Genetic Origin	
	CHB	CHW	MLN	MRC	VLD	HYB	TRD
Egg Weight (g)	62.11 ^a^ ± 1.106	58.00 ^ab^ ± 1.43	59.47 ^a^ ± 1.19	33.97 ^c^ ± 1.29	51.91 ^b^ ± 1.92	60.57 ^a^ ± 2.00	48.50 ^b^ ± 1.82
Albumen (g)	39.64 ^a^ ± 0.842	35.21 ^b^ ± 1.09	30.10 ^c^ ± 0.90	14.32 ^d^ ± 0.98	29.42 ^c^ ± 1.46	37.98 ^a^ ± 1.36	24.00 ^b^ ± 1.24
Yolk (g)	14.24 ^b^ ± 0.404	15.19 ^b^ ± 0.52	20.84 ^a^ ± 0.43	13.99 ^b^ ± 0.47	15.54 ^b^ ± 0.70	14.60 ^b^ ± 0.59	17.33 ^a^ ± 0.53
Shell (g)	8.23 ^a^ ± 0.192	7.60 ^ab^ ± 0.25	8.53 ^a^ ± 0.21	5.66 ^c^ ± 0.22	6.90 ^b^ ± 0.33	7.99 ^a^ ± 0.25	7.17 ^b^ ± 0.23
Edible part (g)	53.89 ^a^ ± 1.030	50.40 ^ab^ ± 1.33	50.94 ^ab^ ± 1.11	28.31 ^c^ ± 1.20	44.96 ^b^ ± 1.78	52.58 ^a^ ± 1.80	41.33 ^b^ ± 1.64
Albumen (%)	63.77 ^a^ ± 0.712	60.66 ^ab^ ± 0.92	50.51 ^c^ ± 0.76	42.14 ^d^ ± 0.83	56.58 ^b^ ± 1.23	62.60 ^a^ ± 1.01	48.38 ^b^ ± 0.92
Yolk (%)	22.96 ^e^ ± 0.581	26.20 ^d^ ± 0.75	35.10 ^b^ ± 0.62	41.15 ^a^ ± 0.68	30.05 ^c^ ± 1.01	24.17 ^b^ ± 0.79	36.52 ^a^ ± 0.72
Shell (%)	13.27 ^b^ ± 0.359	13.14 ^b^ ± 0.46	14.39 ^b^ ± 0.39	16.70 ^a^ ± 0.42	13.37 ^b^ ± 0.62	13.22 ^b^ ± 0.34	15.09 ^a^ ± 0.31
Edible part (%)	86.73 ^a^ ± 0.359	86.86 ^a^ ± 0.46	85.61 ^a^ ± 0.39	83.30 ^b^ ± 0.42	86.63 ^a^ ± 0.62	86.77 ^a^ ± 0.34	84.91 ^b^ ± 0.31

Commercial Hybrid Brown (CHB), Commercial Hybrid White (CHW), Milanino (MLN), Mericanel della Brianza (MRC), Valdarnese Bianca (VLD). LS means within a row lacking a common letter differ significantly: ^a,b,c,d,e^ (*p* ≤ 0.05).

**Table 3:** Yolk fatty acids composition (%; LS mean ± SE) by breed.

	Breeds					Genetic Origin	
	CHB	CHW	MLN	MRC	VLD	HYB	TRD
C16:0	24.76 ^bc^ ± 0.25	25.74 ^b^ ± 0.32	25.11 ^b^ ± 0.27	23.81 ^c^ ± 0.29	27.92 ^a^ ± 0.43	25.13 ± 0.30	25.10 ± 0.27
C16:1n7	0.92 ^c^ ± 0.14	1.41 ^c^ ± 0.18	3.11 ^a^ ± 0.15	2.21 ^b^ ± 0.16	3.22 ^a^ ± 0.24	1.10 ^b^ ± 0.13	2.79 ^a^ ± 0.12
C18:0	9.05 ^b^ ± 0.33	9.82 ^b^ ± 0.43	8.53 ^b^ ± 0.36	10.32^a^ ± 0.39	8.53 ^b^ ± 0.58	9.34 ± 0.29	9.21 ± 0.27
C18:1n9	42.89 ^a^ ± 0.54	38.63 ^b^ ± 0.69	40.24 ^b^ ± 0.58	38.51 ^b^ ± 0.63	39.98 ^b^ ± 0.94	41.29 ^a^ ± 0.52	39.54 ^b^ ± 0.47
C18:2n6	14.34 ^bc^ ± 0.46	17.61 ^a^ ± 0.60	16.10 ^ab^ ± 0.50	16.64 ^a^ ± 0.54	13.26 ^c^ ± 0.80	15.57 ± 0.46	15.82 ± 0.42
C20:4n6	2.71 ^ab^ ± 0.11	2.32 ^b^ ± 0.14	2.59 ^b^ ± 0.12	2.95 ^a^ ± 0.13	1.71 ^c^ ± 0.19	2.57 ± 0.11	2.57 ± 0.10
C22:6n3	1.25 ^ab^ ± 0.11	1.17 ^ab^ ± 0.15	0.87 ^a^ ± 0.12	1.01 ^ab^ ± 0.13	1.62 ^b^ ± 0.20	1.22 ± 0.10	1.06 ± 0.09
SFA *	34.54 ^ba^ ± 0.40	36.12 ^a^ ± 0.52	34.14 ^b^ ± 0.43	34.68 ^b^ ± 0.47	37.22 ^a^ ± 0.70	35.13 ± 0.37	34.88 ± 0.34
MUFA	46.23 ^a^ ± 0.55	41.35 ^bc^ ± 0.72	43.71 ^b^ ± 0.60	41.07 ^c^ ± 0.65	43.46 ^abc^ ± 0.96	44.40 ^a^ ± 0.57	42.67 ^b^ ± 0.52
PUFA *	19.08 ^b^ ± 0.61	22.48 ^a^ ± 0.79	22.14 ^a^ ± 0.66	24.24 ^a^ ± 0.71	18.69 ^b^ ± 1.06	20.35 ^b^ ± 0.60	22.34 ^a^ ± 0.55
n6/n3 *	13.36 ^a^ ± 0.85	14.51 ^a^ ± 1.10	12.57 ^a^ ± 0.91	11.50 ^a^ ± 0.99	5.26 ^b^ ± 1.47	13.79 ^a^ ± 0.80	10.91 ^b^ ± 0.70
AI	0.40 ^bc^ ± 0.01	0.42 ^b^ ± 0.01	0.39 ^bc^ ± 0.01	0.38 ^c^ ± 0.01	0.48 ^a^ ± 0.01	0.41 ± 0.01	0.40 ± 0.01
TI	0.94 ± 0.02	0.99 ± 0.03	0.88 ± 0.02	0.88 ± 0.03	0.96 ± 0.04	0.96 ^a^ ± 0.02	0.89 ^b^ ± 0.02

Commercial Hybrid Brown (CHB), Commercial Hybrid White (CHW), Milanino (MLN), Mericanel della Brianza (MRC), Valdarnese Bianca (VLD). SFA = Saturated Fatty Acids, MUFA = Monounsaturated Fatty Acids, PUFA = Polyunsaturated Fatty Acids n6/n3 ratio; Atherogenic Index (AI) and Thrombogenic Index (TI). LS means within a row lacking a common letter differ significantly: ^a,b,c^, (*p* ≤ 0.05).
